# RT-PCR–DGGE Analysis to Elucidate the Dominant Bacterial Species of Industrial Spanish-Style Green Table Olive Fermentations

**DOI:** 10.3389/fmicb.2016.01291

**Published:** 2016-08-17

**Authors:** Antonio Benítez-Cabello, Joaquín Bautista-Gallego, Antonio Garrido-Fernández, Kalliopi Rantsiou, Luca Cocolin, Rufino Jiménez-Díaz, Francisco N. Arroyo-López

**Affiliations:** ^1^Food Biotechnology Department, Instituto de la Grasa, Agencia Estatal Consejo Superior de Investigaciones CientíficasSeville, Spain; ^2^Dipartimento di Scienze Agrarie, Forestali e Alimentari, Agricultural Microbiology and Food Technology Sector, University of TorinoTorino, Italy

**Keywords:** bacterial biodiversity, biofilms, *Lactobacillus*, marine salt, RT-PCR-DGGE, table olives

## Abstract

This paper describes the dominant bacterial species metabolically active through the industrial production of Spanish-style Manzanilla and Gordal olives. For this purpose, samples (brines and fruits) obtained at 0, 15, and 90 fermentation days were analyzed by a culture-independent approach to determine viable cells by reverse transcription of RNA and further PCR-DGGE analysis, detecting at least 7 different species. *Vibrio vulnificus, Lactobacillus plantarum* group, and *Lactobacillus parafarraginis* were present in samples from both cultivars; *Lactobacillus sanfranciscensis* and *Halolactobacillus halophilus* were detected only in Gordal samples, while *Staphylococcus* sp. was exclusively found at the onset of Manzanilla fermentations. Physicochemical data showed a typical fermentation profile while scanning electron microscopy confirmed the *in situ* biofilm formation on the olive epidermis. Different *Bacillus, Staphylococcus*, and *Enterococcus* species, not detected during the fermentation process, were also found in the solid marine salt used by the industry for preparation of brines. Elucidation of these non-lactic acid bacteria species role during fermentation is then an appealingly challenge, particularly regarding safety issues.

## Introduction

The Spanish-style green olive fermentation is probably the most appreciated and popular elaboration of table olives, with approximately 60% of the worldwide table olive production. Its processing is characterized by the use of a sodium hydroxide solution (20–25 g/l) for debittering of fruits. Then, olives are washed for 6–16 h to remove the excess of alkali and brined (110–120 g salt/L). After that fruit usually undergo spontaneous fermentation (Garrido Fernández et al., 1997). The coexistence of diverse fungal (mainly yeasts) and bacterial [lactic acid bacteria (LAB), *Enterobacteriaceae* and *Propionobacteriaceae*] species during fermentation has been abundantly reported. The importance of these microbial groups on the quality, safety and organoleptic profile of the final products is also well known (Garrido Fernández et al., 1997; Arroyo-López et al., 2012a; Hurtado et al., 2012).

The molecular study of the microbial biodiversity present during table olive processing can be performed either by culture-dependent or by culture-independent methods (Botta and Cocolin, 2012). The molecular approach based on the cultivation of microorganisms and further isolation of DNA or RNA obtained from the cells do not offer a complete profile of the microbial diversity present in the ecosystem (Hugenholtz et al., 1998; Botta and Cocolin, 2012; Cocolin et al., 2013a). It is estimated that above 90% of the microorganisms from natural environments and approximately 25–50% of those present in fermented foods cannot be cultivated, using conventional microbiological techniques (Amann and Kühl, 1998; Ampe et al., 1999; Cocolin et al., 2013a). In food microbiology, molecular culture-dependent techniques are being extensively used either for identification (RFLP ITS-5.8S rRNA gene, sequencing 16S and 26S rRNA genes, multiplex PCR assay of the *recA* gene) or typing (RAPD-PCR, rep-PCR, PFGE) of microorganisms (Botta and Cocolin, 2012).

On the contrary, PCR-DGGE (Denaturing Gradient Gel Electrophoresis) is a very useful culture-independent method where the genetic material is directly obtained from food samples without cultivation of the microorganisms. In table olives, the use of PCR-DGGE analysis has been used mainly to study the microbial diversity present in brines (Abriouel et al., 2011; Muccilli et al., 2011; Randazzo et al., 2012; Cocolin et al., 2013b; Tofalo et al., 2014; Lucena-Padrós et al., 2015). However, PCR-DGGE does not discern between viable and non-viable microorganisms since DNA can also proceed from dead cells (Keer and Birch, 2003; Cenciarini-Borde et al., 2009). On the contrary, reverse transcription (RT) of RNA to cDNA provides an estimation exclusively of viable microorganisms with metabolic activity (Santarelli et al., 2008). Few authors have used techniques involving the use of RNA in the study of table olives microbiota (Cocolin et al., 2013b; De Angelis et al., 2015). Bearing in mind that olives are finally ingested by consumers, the analysis of the microbiota adhered to fruits forming biofilms is also critical (Nychas et al., 2002; Arroyo-López et al., 2012b; Domínguez-Manzano et al., 2012; Grounta and Panagou, 2014; Benítez-Cabello et al., 2015) albeit, in this case, the studies are still scarce. Only recently, Cocolin et al. (2013b) and De Angelis et al. (2015) have used a metagenomics-based independent-culture approach for the study of the bacterial biodiversity adhered to olive surfaces.

This survey aims to study the bacterial biodiversity present in industrial Spanish-style green table olive fermentations, using RT-PCR-DGGE analysis for the identification of the predominant species metabolically active during fermentation of Gordal and Manzanilla olives. Also, the paper analyses, for the first time, the viable bacterial species present in the solid marine salt, obtained from the saltworks of the Atlantic coast of Cádiz (Spain), used by the industry to prepare the fermentation brines.

## Materials and Methods

### Industrial Process and Sample Collection

The fruits (Manzanilla and Gordal varieties) were debittered with a NaOH solution (3.2 and 2.5%, respectively, according to the specific characteristics of olive varieties) and washed for 6 h to remove excess of alkali. Then, the olives were transferred into the industrial fermentation vessels (9.700 kg of fruits and 5.900 L, brine, making a total volume of, approximately, 16,000 L) and brined (11%, wt/vol, sea salt solution) following by a spontaneous fermentation. Brine (50 mL) or olive (25 g) samples were obtained from duplicated fermentation vessels at 0, 3, 6, 10, 15, 20, 30, 64, and 90 days of processing, during 2013/2014 season. Samples were labeled as G35 and G36 (for Gordal) and M1371 and M1372 (for Manzanilla), and transported in refrigerated conditions to the laboratory for their processing in the same day.

### Monitoring of the Fermentations

Physicochemical control of the fermentation brines was achieved through periodical determination of pH, NaCl concentration (%, wt/vol), titratable (expressed as lactic acid, g/100 mL) and combined acidities (expressed as undissociated organic salts, Eq/L), according to the methods described by Garrido Fernández et al. (1997).

The microbial populations adhered to fruits were studied by washing olives twice in phosphate buffer solution (PBS), to remove non-adhered cells, pitting and weighting the olives and immediately transferring (~10 g) into a stomacher bag containing 75 mL of a sterile saline solution (0.9%, NaCl). Then, the fruits were homogenized for 1 min at 300 rpm in a stomacher model Seward 400 (Seward Medical, Ltd., West Sussex, England). Suspensions of the samples or appropriated dilutions were then plated onto solid culture media using a Spiral System (model dwScientific, Don Whitley Scientific Limited, England). *Enterobacteriaceae* were counted on Crystal Violet Neutral-Red Bile Glucose (VRBD) agar (Merck, Darmstadt, Germany), LAB were spread onto de Man Rogosa and Sharpe (MRS) agar (Oxoid, Basingstoke, Hampshire, England) supplemented with 0.02% (wt/vol) sodium azide (Sigma, St. Luis, MI, USA), and yeasts were grown on yeast-malt-peptone-glucose medium (YM) agar (Difco, Becton and Dickinson Company, Sparks, MD, USA) supplemented with oxytetracycline and gentamicin sulfate (0.005%, wt/vol) as selective agents. The plates were incubated at 30°C for 48–72 h, counted using a Flash & Go (IUL, Barcelona, Spain) image analysis system and expressed as log_10_ CFU/g.

Samples obtained from fermentation brines were diluted, if necessary, in sterile saline solution (0.9% NaCl) and plated on the culture media described above. After incubation as described for fruit samples, counts in brines were expressed as log_10_ CFU/mL.

### Control of the Biofilm Formation on the Olive Skin

At different stages of fermentation (0, 15, and 90 days), the presence of biofilms on the epidermis of fruits was assessed by scanning electron microscopy (SEM), as described by Kroupitski et al. (2009) with slight modifications. First, fruits were rinsed twice for 15 min in PBS to remove the non-adhered cells and then fixed in 2.5% glutaraldehyde (Sigma–Aldrich, St. Louis, MI, USA) in PBS for 2.5 h. Later, the olives were dehydrated through a graded ethanol series (50, 70, 80, 90, 95, and 100%, 5 min in each one). Finally, fruits were treated for 20 min in 2-methyl-2-propanol. For SEM observation, 2 mm × 2 mm slices of the skin of olives were taken, placed on glass slides, and coated with gold in a Scancoat Six SEM sputter coater (Edwards, Crawley, England). Pictures were taken with a JEOL JSM- 6460LV SEM model (JEOL USA, Inc., Peabody, MA) in the Technology and Innovation Research Center (CITIUS, University of Seville, Spain).

### RNA Extraction and Reverse Transcription (RT)

To investigate metabolically active microorganisms growing in both brines and the skin of olives, RT of RNA samples taken at 0, 15, and 90 days of fermentation were performed. One mL of brine or 1 mL of the homogenate of olives were centrifuged (13,000 rpm, 10 min, 4°C), 150 μL of RNA LATER (Ambion, AH7021H) were added to the resulting pellet, and samples were stored at –80°C until use. Sample preparation for RNA extraction was performed according to the protocol reported by Rantsiou et al. (2008). Three μL of TURBO-DNase (Ambion, Milan, Italy) was added to digest the DNA in the samples (3 h, 37°C). The presence of residual DNA was checked by PCR (Cocolin et al., 2001). Finally, RT-PCR was performed using the universal primers 338f (5′-ACTCCTACGGGAGGCAGCAGCAG-3′) and 518r (5′-ATTACCGCGGCTGCTGG-3′) (Ampe et al., 1999), which anneal to the variable V3 region of the 16S rRNA bacterial gene (Cocolin et al., 2013b).

### DGGE and Cluster Analysis

The different amplicons obtained from RT-PCR were analyzed by DGGE with a Dcode universal mutation detection system (BioRad, Milan, Italy), according to the protocol described by Dolci et al. (2008). For this purpose, a gradient from 40 to 60% of polyacrylamide (acrylamide- bis acrylamide 37:5:1, 8% wt/vol) was used. Electrophoresis was conducted at 200 V for 5 h (with an initial step of 10 min at 80 V) at 60°C in TAE buffer (×1) (10 mmol/L Tris-borate, 1 mmol/L, EDTA, pH 8.0). Gels were stained for 20 min in TAE buffer (×1) containing 1X SYBR Green I (Sigma) and then analyzed under UV using UVI pro platinum 1.1 Gel Software (Eppendorf, Hamburg, Germany). Selected DGGE bands were excised from the gel with sterile pipette tips and purified in water. One microliter of the eluted DNA was used for the re-amplification using the primers and the conditions described above, and the PCR products were checked using DGGE and sent for sequencing to MWG Biotech (Dolci et al., 2008). Partial sequences from the 16S rRNA gene were then aligned with previous sequences deposited in GenBank database using the Blast tool program to determine the closest known relative species (Altschul et al., 1997). Finally, similarities between the bacterial community profiles generated by RT-PCR-DGGE analysis from different samples were determined by clustering analysis. For this purpose, the banding profiles were normalized and analyzed with the BioNumerics 6.6 software package (Applied Maths, Kortrijk, Belgium). The DICE correlation coefficient and the UPGMA clustering algorithm (means of the unweighted pair group method with arithmetic averages) was used to calculate the similarities among DGGE patterns and to obtain the dendrograms.

### Study of the Solid Marine salt by Culture-Dependent Molecular Methods

Ten grams of the commercial sea salt, used to prepare the fermentation brines and purchased by the industry in the salt plant of Puerto de Santa Maria (Atlantic coast of Cádiz, Spain), were diluted into 50 mL of sterile saline water (0.9% NaCl) and spread on plate count agar (PCA) (Oxoid, Basingstore, Hampshire, UK) for determination of aerobic mesophilic microorganisms. Plates were incubated at 30°C for 24 h. Then, a total of 8 colonies with different morphology were taken and purified for identification purposes using universal primers 27F (5′-AGAGTTTGATCCTGGCTCAG-3′) and 1492R (5′-GGTTACCTTGTTACGACTT-3′) targeted for the small-subunit 16 rRNA gene of bacteria (Barrangou et al., 2002). Analyses were carried out in duplicate.

## Results and Discussion

### Physicochemical Control of Fermentation

**FIGURE 1:**
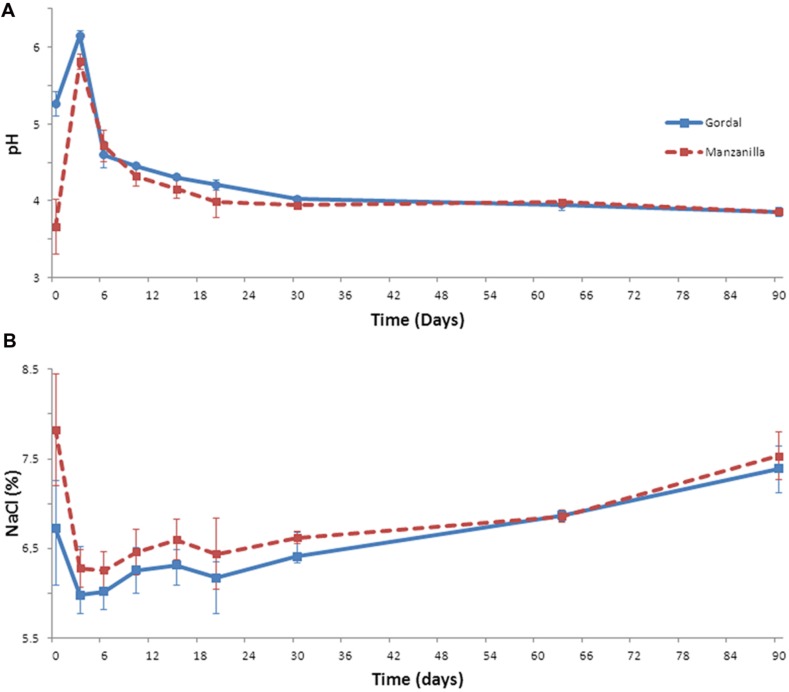
**Changes of pH (A) and salt (B) through industrial Spanish-style fermentations of Gordal and Manzanilla olives**.

The evolution of the fermentation process of the Gordal and Manzanilla olives was followed by recording the physicochemical data through a total of 90 days from duplicate industrial fermentation vessels. The proper changes in pH and salt are essential (together with titratable acidity), to ensure the microbial safety of the fermented olives and to control the growth of spoilage and pathogen microorganisms during fermentation (Perricone et al., 2010). In this experiment, after fruits’ brining, pH increased rapidly during the first 3 days from an initial value of 5.3 to 6.2 in the case of Gordal olives, and from 3.7 to 5.8 in the case of Manzanilla fermentations (**Figure 1A**). This increase in pH during the initial stages of industrial lye-treated olive processing is usual and due to: (i) the diffusion into the flesh of the organic acids initially added to the cover brine, and (ii) the leaching of residual sodium hydroxide from the pulp into the brines (Garrido Fernández et al., 1997). From this moment onward, the pH decreased quickly. In both cases, the equilibrium between the olive flesh and cover brine was reached approximately at day 30 (around 3.8 units), and it was kept constant until the end of the fermentation process. Regarding salt concentration (**Figure 1B**), it was observed a decrease during the first 3 days from the initial 7.8 to 6.2, in the case of Manzanilla olives, and from 6.7 to 5.9% in the case of Gordal fermentations. As in the pH, this phenomenon is due to equilibrium processes between the cover brines and fruits (Garrido Fernández et al., 1997). Then, in both fermentations systems, a slight increase through the fermentative process was noticed due to replacement of lost liquid with new brine, obtaining a final salt concentration around 7.5% at 90 days.

**FIGURE 2:**
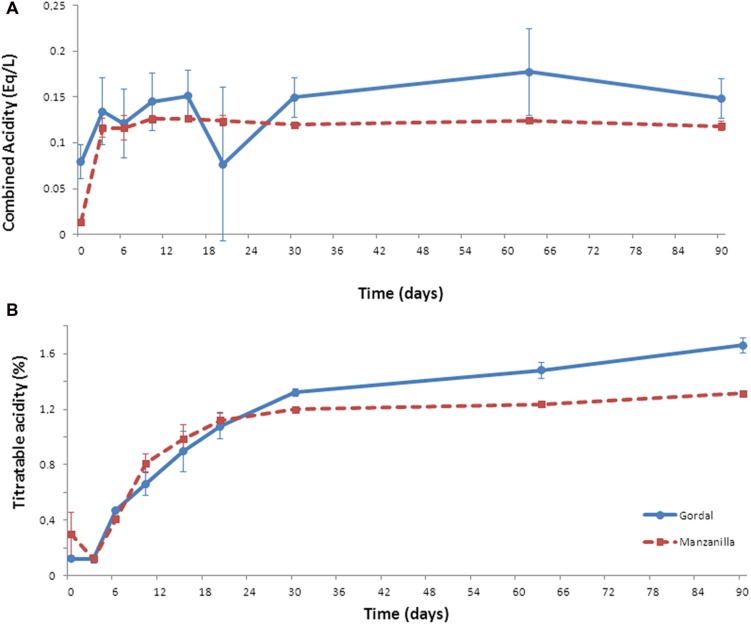
**Changes of combined (A) and titratable (B) acidities through industrial Spanish-style fermentations of Gordal and Manzanilla olives**.

Combined acidity increased throughout the fermentation process from the initial 0.08 to final 0.15 Eq/L in Gordal fermentations, and from initial 0.01 to final 0.12 Eq/L in the case of Manzanilla olives (**Figure 2A**). For titratable acidity, this parameter increased from day 3 onward coinciding with the beginning of the LAB growth (see below). The increase of this parameter during the fermentation process is due to the production of lactic acid by the activity of LAB (Garrido Fernández et al., 1997; Hurtado et al., 2012). A higher final value was obtained in the case of Gordal (1.4%) than Manzanilla (1.2%) fermentations (**Figure 2B**).

These changes in pH and salt, together with the combined and titratable acidities obtained, are typical of this kind of table olive fermentations (Garrido Fernández et al., 1997). Furthermore, the pH value far below the limit established for Spanish-style olive (<4.3) in the Table Olive Standard (International Olive Oil Council [IOC], 2004) and the titratable acidity value above 1.0%, are important aspects to ensure product safety (Garrido Fernández et al., 1997; International Olive Oil Council [IOC], 2004). Hence, both (Gordal and Manzanilla) Spanish-style fermentations followed an adequate fermentation process from the physicochemical point of view. Thus, the bacterial biodiversity found could be considered as a good representation of this type of process.

### Microbiological Control of Fermentation

By plate counts, *Enterobacteriaceae* were never detected during the 90 days of fermentation, in either Gordal or Manzanilla olives (data not shown). As mentioned above, low pH levels exert a considerable inhibitory effect on this microbial group (Garrido Fernández et al., 1997). Yeasts were detected at the onset of fermentation with population levels around 4 log_10_ CFU/g (in fruits) or 3 log_10_ CFU/ml (in brines). These microorganisms disappeared from the epidermis of the fruits through the fermentation process, while in brines their presence was most relevant obtaining a maximum count of around 5 log_10_ CFU/mL at 15th day of fermentation (data not shown). Then, the yeasts declined to a population level around 2 log_10_ CFU/mL at the end of the process. As reported by Arroyo-López et al. (2012b), the presence of these microorganisms during Spanish-style green table olive fermentations is usual. Because of the relative low yeast population levels obtained for this microbial group during the experiment (compared to LAB as shown below), they were not included in further molecular analysis.

**FIGURE 3:**
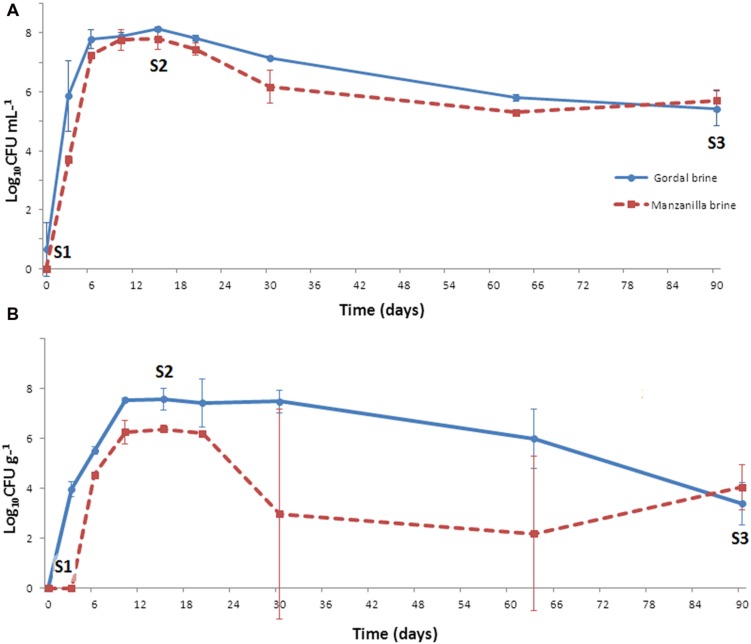
**Changes of LAB population in brine (A) and fruit (B) through industrial Spanish-style fermentations of Gordal and Manzanilla olives**. S1, S2, and S3 stand for the sampling time for RT-DGGE analyses at 0, 15, and 90 days of fermentation, respectively.

On the contrary, the growth of LAB population was considerable in both Gordal and Manzanilla lye-treated olives. At the onset of fermentation, the presence of these microorganisms in either brine or fruits was practically negligible. However, they were able to grow quickly obtaining at the 15 day of fermentation a maximum population level of approximately 8 log_10_ CFU/ml (in brines) or 7.6 (Gordal) and 6.4 log_10_ CFU/g (Manzanilla) in fruits (**Figure 3**). From this moment, there was a slight decline in the LAB population, less marked for Gordal fermentations (which justify the higher titratable acidity values obtained compared to Manzanilla). At the 90th day of fermentation, population levels from 3.7 log_10_ CFU/g (fruits) to 5.6 log_10_ CFU/mL (brine) were still noticed. Therefore, in both cases, the fermentations were clearly dominated by LAB, which moreover followed the normal evolution of this type of processes (Garrido Fernández et al., 1997; Hurtado et al., 2012).

### Biofilm Formation

**FIGURE 4:**
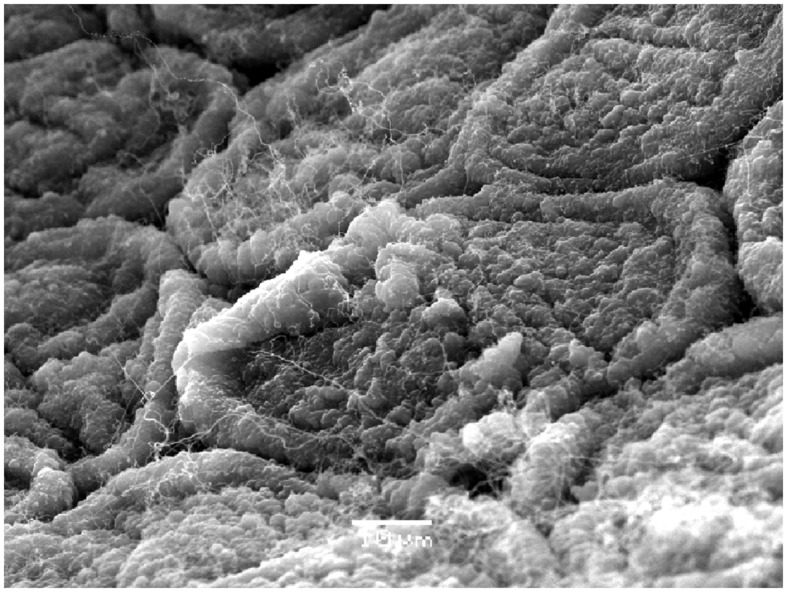
**Scanning electron microscopy (SEM) picture showing the formation of a biofilm (15th day) on the surface of Manzanilla fruits fermented according to Spanish-style**.

Scanning electron microscopy pictures (**Figure 4**) provide a clear evidence of the capacity of microorganisms to aggregate and colonize the olive surface during the fermentation process. In the figure, it is clearly distinguishable the form of the olive cells as well as the first stages of biofilm formation (the photograph was taken at 15th day of fermentation), the microorganisms attached to the olive epidermis, and the production of the exopolysaccharide’ matrix. Nychas et al. (2002) first reported the presence of both LAB and yeast populations colonizing the epidermis of directly brined olives. More recently, the formation of true mixed biofilms during Spanish-style green table olive fermentations was reported for different types of olive varieties by Arroyo-López et al. (2012a) and Domínguez-Manzano et al. (2012). Benítez-Cabello et al. (2015) and Grounta and Panagou (2014) have also shown, by SEM, the formation of biofilms on Greek black oxidized and directly brined Gordal olives, respectively.

### Analysis of the Bacterial Biodiversity through Fermentation by RT-PCR-DGGE

*Enterobacteriaceae*, yeasts, and LAB are considered the most relevant group of microorganisms with influence during table olive processing (Garrido Fernández et al., 1997; Arroyo-López et al., 2012a; Hurtado et al., 2012). Thereby, brines are usually plated on selective media for these microbial groups to determine the proper evolution of the fermentation process. However, other microorganisms present during fermentation could be missing because of this selective culture-dependent approach. In fact, when olive samples are also plated on other selective media for *Pseudomonas, Clostridium*, or *Staphylococcus*, it is possible to detect the presence of these microorganisms in the fermentation environment (Nychas et al., 2002; Lucena-Padrós et al., 2014). For these reasons, in this study, a culture-independent molecular approach (RT-PCR-DGGE) to determine the evolution of the main bacterial species during fermentation of Gordal and Manzanilla varieties, processed according to green Spanish-style (lye treated olives), was adopted. Apparently, with this approach only large populations (>10^3^ CFU/mL) are detected (Murray et al., 1996; Cocolin et al., 2001). Because of the previous reverse transcription of the RNA samples to cDNA (RT-PCR), a vision of the metabolically active bacterial groups was obtained.

**FIGURE 5:**
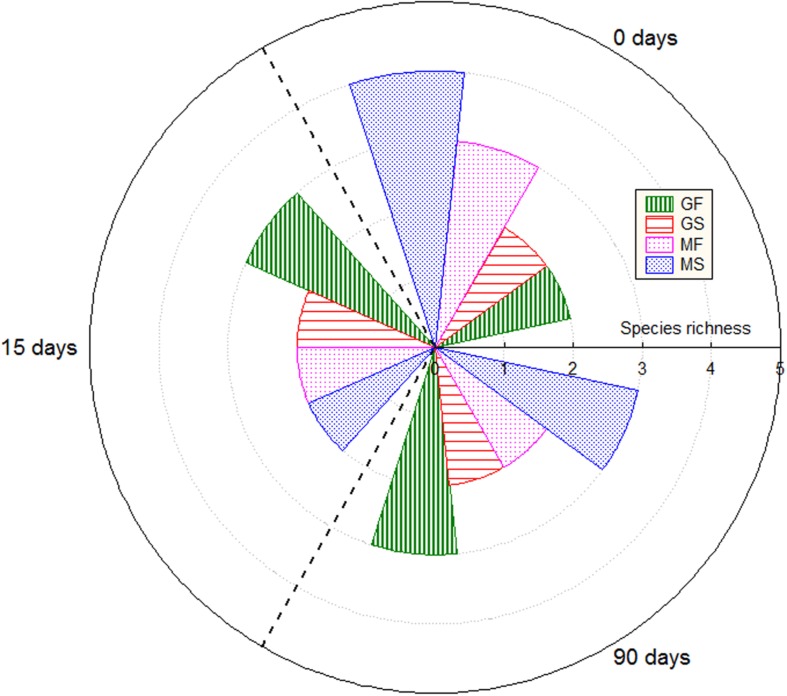
**Species richness through fermentation process for the different types of samples**. GF (Gordal fruits), GS (Gordal brines), MF (Manzanilla fruits), and MS (Manzanilla brines).

The DGGE analysis of the samples of brines and fruits obtained during fermentation of Gordal olives revealed a low bacterial biodiversity. Six different DGGE bands were obtained including both duplicate fermentation vessels. After sequencing and Blast search, they were assigned to *Lactobacillus sanfranciscensis, Halolactibacillus halophilus, Vibrio vulnificus, Lactobacillus parafarraginis, Lactobacillus plantarum* group, and *Vibrio* sp., in all cases with a high percentage of identity (>98%) (**Table 1**). Albeit other two bands were also obtained from DGGE gels, they could not be unequivocally identified for any species. One was assigned to an uncultured bacterium (closest accession number in NCBI: KF325061.1) while the other was related to *Chroococcidiopsis thermalis*, an extremophile photosynthetic cyanobacteria. Because of the small percentage of identity obtained in this last case (92%), it is very probable that the universal pairs of primers used (338f and 518R) were unspecific to bacterial 16S rRNA. Thereby, they could also have amplified a partial sequence of chloroplast DNA. It is an aspect to confirm, but the cyanobacteria origin of the chloroplast organelle in vegetable cells it is well argued (Bonen and Doolittle, 1975). In the case of Manzanilla fermentations, a low biodiversity was also noticed, with only 4 different bands detected plus other 2 corresponding to the uncultured bacterium and the presumptive chloroplast DNA (**Table 1**). The assigned bands in Manzanilla fermentations corresponded to *L. plantarum* group*, V. vulnificus, L. parafarraginis*, and *Staphylococcus* sp., with a percentage of identity higher than 99% (except for *Staphylococcus*, which was 96%). The species richness (**Figure 5**) through the fermentation process for each type of sample ranged from 2 to 4 in the best of the cases (obtained for samples of Manzanilla brines at 0 days). Including in the analysis all samples obtained from the Gordal and Manzanilla Spanish-style fermentations, a total number of 7 different bacterial species were found (**Table 1**). This biodiversity is low in comparison to those reported in previous studies, in which the total number of species ranged from to 10 to 17 (Abriouel et al., 2011; Cocolin et al., 2013b; Tofalo et al., 2014; Lucena-Padrós et al., 2015). Maybe the difference could be due to the different approach used in this study, which only allow the identification of the predominant bacteria species with metabolic activity due to reverse transcription of RNA samples (RT-PCR).

**Table 1:** Bacterial species identification after sequencing of the variable V3 region of the 16S rRNA gene purified from PCR-DGGE profiles obtained from reverse transcription of RNA directly extracted from Gordal and Manzanilla samples.

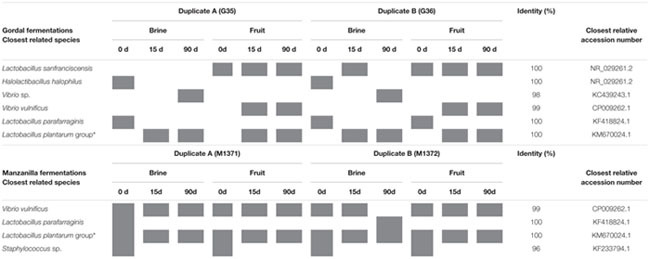

*^∗^Include the species Lactobacillus plantarum, Lactobacillus pentosus, and Lactobacillus paraplantarum, which were indistinguishable using this technique.*

The role played by *L. plantarum* and *L. pentosus* during table olive fermentations is crucial because of the production of lactic acid and bacteriocins, which contributes to safeguard olives from spoilage and pathogen microorganisms (Hurtado et al., 2012). Both species are genotypically and phenotypically closely related. In fact, the sequencing of the 16S rRNA fragment cannot discriminate between them and *Lactobacillus paraplantarum* (Botta and Cocolin, 2012). The sequence assigned to the *L. plantarum* group was obtained practically through all fermentation process, either in brine or fruits, indicative of the good adaptation of this microorganism to the fermentation environment. Furthermore, the ability of *L. pentosus* and *L. plantarum* to dominate olive fermentation and colonize fruit surface, forming biofilms, has recently been reported (Arroyo-López et al., 2012b; Domínguez-Manzano et al., 2012; Hurtado et al., 2012; Cocolin et al., 2013b; Grounta and Panagou, 2014; Tofalo et al., 2014; Benítez-Cabello et al., 2015).

*V. vulnificus* was also detected in many samples through the fermentation process in both Gordal and Manzanilla (brines and fruits). Previous PCR-DGGE studies have also shown the presence of *Vibrio* sp. during olive fermentations (Abriouel et al., 2011; Lucena-Padrós et al., 2015). *Vibrio* is a genus of halophilic Proteobacteria, which includes several species associated with human gastroenteritis diseases. In recent years, there is a constant increase worldwide of recognized infections caused by pathogenic non-cholera vibrios (Igbinosa and Okoh, 2008). Specifically, *V. vulnificus* can be isolated from foods or human specimens and produce human disease (Austin, 2010). Infections with *V. vulnificus* are often associated with the eating of raw oysters and are the leading cause of seafood-related deaths in the United States (Daniels, 2011). To our knowledge, this is the first time that specifically *V. vulnificus* has been reported in table olive processing. The influence of this microorganism during olive fermentations is unknown, and further studies should be carried out on this issue, especially on the safety aspects.

The DGGE analyses also showed the presence of *L. sanfranciscensis* and *Halolactibacillus halophilus* in Gordal fermentation samples, while *Staphylococcus sp.* was only found in Manzanilla fermentations. *H. halophilus* and *Staphylococcus* were exclusively detected at the onset of fermentation, mainly in brines samples. This fact is indicative that both species do not have influence in the fermentation process. The only reference to the presence of *H. halophilus* in table olives has been recently reported by Lucena-Padrós et al. (2015), while Medina-Pradas and Arroyo-López (2015) mention the presence of *Staphylococcus* in diverse table olive processing. On the contrary, *L. sanfranciscensis* was obtained from the epidermis of the fruit through all fermentation process in Gordal olives. To our knowledge, this is the first time that this heterofermentative LAB, widely used in the sourdough production (De Vuyst et al., 2014), has been reported in table olive fermentations. *L. parafarraginis* was identified from both Gordal and Manzanilla fermentations mainly from brine samples at the onset of fermentation. This species was also detected using DGGE analysis in Spanish-style fermentations by Lucena-Padrós et al. (2015), and isolated from Spanish-style olive packaging by Montaño et al. (2013).

**FIGURE 6:**
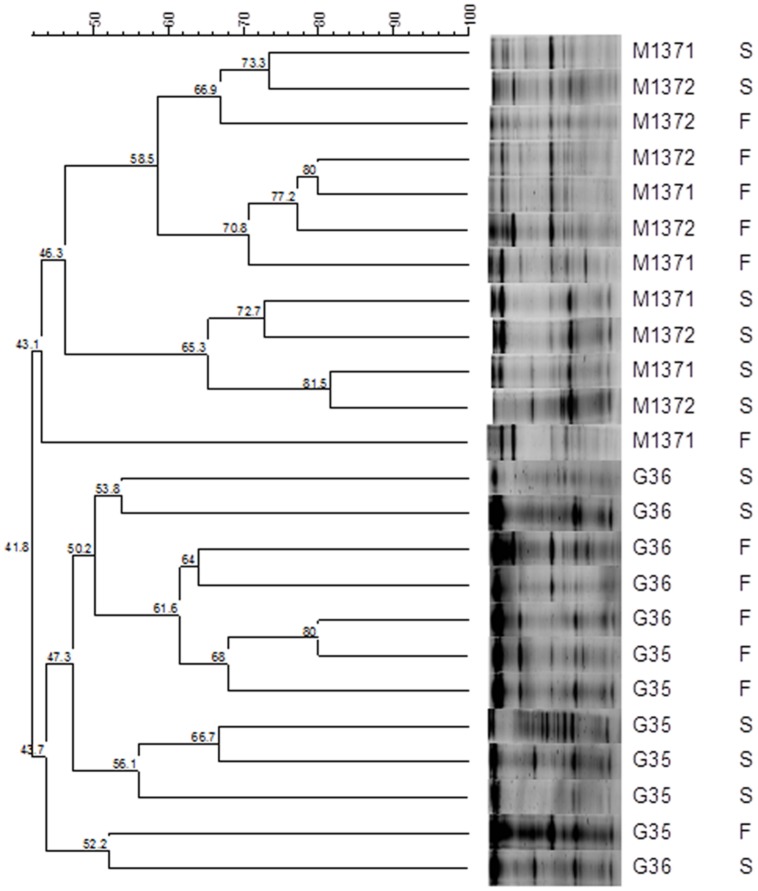
**Dendrogram obtained from comparison of RT-PCR-DGGE profiles by DICE correlation of the olive (*F*) and brine (*S*) samples obtained from Gordal (*G*) and Manzanilla (*M*) fermentations.** Samples were obtained from duplicate fermentation vessels of Manzanilla (M1371 and M1372) and Gordal (G35 and G36) cultivars.

**Figure 6** shows the clustering analysis of the RT-PCR-DGGE profiles obtained with the bacteria universal primers for the different Gordal and Manzanilla samples. The organization of the banding patterns in the dendrogram as a result of the UPGMA method and DICE correlation show a clear trend of grouping by type of olive fermentations (Gordal or Manzanilla). On the contrary, the separation was not affected by the influence of fermentation vessels or type of sample (fruits or brines). A similar methodology was used by Cocolin et al. (2013b) and Lucena-Padrós et al. (2015) to group different DGGE bacterial profiles obtained from olive fermentations as a function of location or type of olive processing.

### Analysis of Bacterial Biodiversity in Marine Salt

**FIGURE 7:**
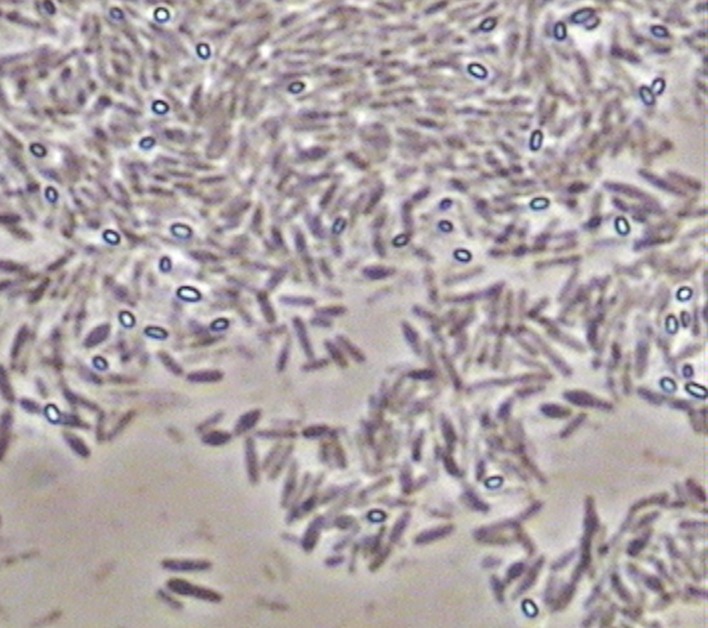
**Picture obtained with a phase contrast microscope (×4000) showing the presence of bright oval endospores in isolates previously identified as *Bacillus* species**.

As commented above, samples of solid marine salt used by industry to prepare fermentation brine were directly plated on PCA medium after the previous dilution with a sterile saline solution. The counts obtained for aerobic mesophilic microorganisms after 24 h of incubation were low (2.79 ± 0.14 log_10_ UFC/g). Then, different isolates were purified and identified by sequencing and further Blast analysis of the small-subunit 16 rRNA gene of bacteria. Diverse *Bacillus* species (*B. drentensis, B. asahii, B. flexus, B. selenatarsetatis, B. alcalophilus*, and *B. alkalisediminis*), as well as the presence of *Staphylococcus epidermis* and *Enterococcus faecium*, were detected in the marine salt samples with a homology higher than 97% (**Table 2**). *Bacillus* is a genus of Gram-positive bacteria ubiquitous in nature, obligate aerobes or facultative anaerobes. Under stressful environmental conditions, the bacteria can produce oval endospores. For this reason, *Bacillus* species were also observed with a phase contrast microscope, always noticing the presence of spores (**Figure 7**). For all species found in marine salt, only *Staphylococcus* sp. was detected by RT-PCR-DGGE analysis at the onset of the fermentation process, which show the high inhibition suffered by both *Bacillus* and *Enterococcus* species during fermentation of Spanish-style green table olives. The protocol used for isolation of microorganisms from salt samples have favored the presence of aerobic mesophilic microorganisms (essentially bacilli), although other halophilic species could be also retrieved by the use of a more selective media enriched in salt.

**Table 2:** Microbial isolates obtained from marine salt and subjected to molecular identification by culture-dependent methods.

Isolate reference	^∗^Matching nucleotides/ identity	^∗∗^Closest related species
TOMC MS1	855 bp | 99%	*Bacillus drentensis* |gi|828177967| KP407110.1
TOMC MS2	694 bp | 99%	*Bacillus asahii* |gi|238800438|gb| FJ973525.1
TOMC MS3	704 bp | 100%	*Staphylococcus epidermis* |gi|955475110| KT633374.1
TOMC MS4	624 bp | 100%	*Bacillus flexus* |gi|954050805| KT720056.1
TOMC MS5	709 bp | 99%	*Bacillus selenatarsenatis* |gi|309253939| HQ202857.1
TOMC MS6	794 bp | 97%	*Bacillus alcalophilus* |gi|343965881| JN540804.1
TOMC MS7	948 bp | 100%	*Enterococcus faecium* |gi|946576338| KR909902.1
TOMC MS8	934 bp | 99%	*Bacillus alkalisediminis* |gi|194239250| AM051268.2

*^∗^Sequence identity of the small-subunit the 16S rRNA gene for bacteria with 27F and 1492R primers. ^∗∗^Accession number for nucleotide sequences and closest related species found in the NCBI GenBank database*.

## Conclusions

RT-PCR-DGGE profiles have revealed a low biodiversity of bacterial species through industrial fermentations of Gordal and Manzanilla olives processed according to Spanish-style. *L. plantarum* group and *V. vulnificus* were the most relevant species because of their presence in all samples obtained from fruits at the end of the fermentation process. On the contrary, the study of marine salt showed a higher biodiversity with the presence of eight different species, many of them belonging to *Bacillus* genera, albeit these microorganisms were not detected during the fermentation process. Data show that these types of studies are necessary to reveal the complex bacterial biodiversity present during table olive fermentations. Also, further studies must also be performed to elucidate the role played by *V. vulnificus* during table olive processing.

## Author Contributions

AB-C, JB-G, FA-L, and KR: performed the experiments, participated in the acquisition, analysis and interpretation of the data, approved the final version of the paper. LC, AG-F, and RJ-D: supervised the laboratory work, participated in the analysis and interpretation of the data, drafted the manuscript, and approved the final version of the paper.

## Conflict of Interest Statement

The authors declare that the research was conducted in the absence of any commercial or financial relationships that could be construed as a potential conflict of interest.
